# A wheat‐specific proline‐rich protein regulates grain weight through a ubiquitin‐mediated delay of endosperm cellularization

**DOI:** 10.1111/tpj.71148

**Published:** 2026-10-10

**Authors:** Li Guo, Yang Yuan, Shuanglin Du, Duoduo Hou, Zongyuan Guo, Shirong Liu, Li Ma, Quan Xie, Zhengqiang Ma, Haiyan Jia

**Affiliations:** ^1^ The Applied Plant Genomics Laboratory of Crop Genomics and Bioinformatics Centre and State Key Laboratory of Crop Genetics & Germplasm Enhancement and Utilization Nanjing Agricultural University Nanjing 210095 Jiangsu China; ^2^ Zhongshan Biological Breeding Laboratory No.50, Zhongling Street Nanjing 210014 Jiangsu China

**Keywords:** endosperm cellularization, grain weight, proline‐rich protein, *TaPGW1‐A1*, *Triticum aestivum*

## Abstract

Grain weight is a key determinant of wheat yield, yet the developmental mechanisms that define this trait remain poorly understood. Here, we identify *TaPGW1‐A1*, a wheat‐specific proline‐rich gene that regulates grain weight. *TaPGW1‐A1* is a recently evolved gene found only in wheat. It is highly expressed in early developing grains, and its overexpression delays endosperm cellularization and prolongs the syncytial phase, resulting in an 8% increase in field‐based grain weight. *Ta*PGW1‐A1 protein directly interacts with the ubiquitin receptor *Ta*DSK2a (Dominant Suppressor of KAR2 homolog) *via* its N‐terminal signal peptide, and the protein–protein complex localizes to the cell periphery. Biochemical assays show that *Ta*DSK2a binds both K48‐ and K63‐linked polyubiquitin chains. Loss‐of‐function of *Ta*DSK2a leads to reduced grain size, confirming its essential role in grain development. The E3 ligase *Ta*RGLG2 inhibits the binding of *Ta*PGW1‐A1 to *Ta*DSK2a. Together, these findings define a *Ta*PGW1‐A1–*Ta*DSK2a–*Ta*RGLG2 regulatory module that links developmental timing with ubiquitin‐mediated protein turnover, revealing a previously unrecognized mechanism controlling endosperm cellularization and grain yield in wheat.

## Significance Statement

We identified a wheat‐specific proline‐rich protein, *Ta*PGW1‐A1, that delays endosperm cellularization through direct interaction with the ubiquitin receptor *Ta*DSK2a, thereby promoting increased grain weight. This study uncovers a previously unrecognized regulatory module that mechanistically links developmental timing to ubiquitin‐mediated protein turnover and establishes *Ta*PGW1‐A1 as a promising molecular target for enhancing wheat yield.

## INTRODUCTION

Common wheat (*Triticum aestivum* L., 2*n* = 6× = 42, AABBDD) is a vital global staple crop, providing nearly one‐fifth of the calories consumed by humans (Shiferaw et al., 2013). Improving wheat productivity is therefore crucial for future food security. Grain weight is a key component of grain yield, and it is highly heritable and environmentally stable, making it an ideal target for grain yield improvement (Abdipour et al., 2016). However, progress is constrained by a limited understanding of the molecular mechanisms underlying wheat grain development. As a polygenic trait, grain weight (GW) is governed by multiple quantitative trait loci (QTLs) (Ma et al., 2022).

To date, more than 700 GW‐associated QTLs have been identified across all 21 wheat chromosomes (Yang et al., 2023). Several of them have been finely mapped, including *qKl‐1BL* (Qin et al., 2024) (2.04 Mb), *QTkw‐2D* (Meng et al., 2023) (9.13 Mb), *Qtgw.nau‐4B* (Yang et al., 2023) (0.6 cM), *Qkt.nau‐5A* (Kong et al., 2022) (0.04 cM), *QTgw.saas‐5B* (Ye et al., 2023) (0.6 cM), *QTKW.caas‐5DL* (Song et al., 2022) (3.9 Mb), and *QTgw.cau‐7D* (Chen et al., 2020) (4.4 Mb). However, cloning these QTLs remains challenging due to recombination suppression in specific genomic regions and the inherent complexity of the hexaploid wheat genome, which contains abundant repetitive sequences (~85%) and extensive segmental duplications (International Wheat Genome Sequencing Consortium (IWGSC), 2018).

Significant progress has been made in identifying grain weight‐related genes through reverse genetics and homologous cloning. Reverse genetic approaches have revealed several genes encoding key regulators of metabolism, including signaling components (*TaSnRK2.3* (Miao et al., 2017), *TaSnRK2.9* (Ur Rehman et al., 2019)), carbon partitioning enzymes (*TaCwi‐A1* (Ma et al., 2012), *TaCWI* (Jiang et al., 2015)), and sucrose synthases (*TaSus2* (Jiang et al., 2011), *TaSus1* (Hou et al., 2014)). Homologous cloning has further identified wheat orthologs of rice genes involved in metabolic regulation, such as *TaGS5* (Wang et al., 2015) (serine carboxypeptidase), *TaGW2* (Su et al., 2011) (E3 ubiquitin ligase), *TaCKX6* (Zhang et al., 2012) (cytokinin oxidase/dehydrogenase), *TaTGW6* (Hanif et al., 2015) (indole‐3‐acetic acid glucose hydrolase), and *TaGW8* (Ma et al., 2019) (SQUAMOSA promoter‐binding protein‐like). Comparative studies indicate that the mechanisms governing grain weight are conserved across cereal species, and that these orthologs serve as valuable targets for haplotype analysis and molecular marker development in wheat improvement programs.

Building on these findings, research in rice and other cereals has revealed the molecular mechanisms by which many genes and pathways regulate grain weight, particularly those mediated by the ubiquitin–proteasome system (UPS) (Long et al., 2024; Ren et al., 2023). For example, *OsCKX2‐2* (Tsago et al., 2020) and *TGW6* (Ishimaru et al., 2013) regulate cytokinin and indole‐3‐acetic acid (IAA) levels, respectively, while *GW2* controls cell proliferation through ubiquitin‐mediated protein turnover (Choi et al., 2018). Two ubiquitin receptors modulate grain weight by distinct mechanisms: *AtDA1* restricts the period of cell proliferation to limit final organ size (Li et al., 2008), whereas *OsHDR3* alters cell proliferation in spikelet hulls (Gao et al., 2021). The UBL‐UBA (ubiquitin like‐ubiquitin associated) protein DSK2a recognizes and binds polyubiquitin chains *via* its UBA domain (Lin et al., 2011), and E3 ligases of the Skp1/Cullin/F‐box (SCF) complex target cell‐cycle regulators for degradation (Noir et al., 2015).

Proline‐rich proteins (PRPs) are structural proteins characterized by a high proline content (Showalter, 1993), and their functional diversification is largely determined by spatiotemporal expression patterns. *PdPRP* in poplar is preferentially expressed in the phloem and immature xylem, where it regulates secondary cell wall biosynthesis (Li et al., 2019). *CsPRP4* in citrus shows predominant expression in leaves and modulates starch metabolism (Ma et al., 2020). In Arabidopsis, *AtPRPL1* is mainly expressed in root trichoblasts and controls root‐hair elongation (Boron et al., 2014). *NtProRP1* in tobacco is highly expressed in pollen grains, pollen tubes, and zygotes; its knockdown impairs pollen germination, pollen tube growth, and early embryogenesis (Chen et al., 2014). In rice, *OsGPRP3* expression peaks in grains at 10 days post anthesis, and its knockout lines exhibit reduced grain weight, smaller grain size, and lower storage protein and lipid contents (Wang et al., 2020). Yet, whether any proline‐rich protein functions as a developmental regulator that interfaces with the ubiquitin–proteasome system is unknown.

Here, we identify *TaPGW1‐A1*, a wheat‐specific proline‐rich protein that is highly expressed during early endosperm development and plays a regulatory role in grain weight. This study reveals a previously unrecognized connection between a developmental proline‐rich protein and ubiquitin‐mediated regulation, providing a new framework for understanding how endosperm cellularization and grain weight are developmentally coordinated in cereals.

## RESULTS

### Predominant expression of a previously uncharacterized gene in developing grains

By mining transcriptomic data from 69 wheat samples representing diverse organs and developmental stages of common wheat (Table S1), we identified a group of proline‐rich protein‐encoding genes with elevated expression predominantly in reproductive tissues, particularly in grains at 2 days post anthesis (DPA) (Figure 1a). Among the six early grain‐enriched transcripts, we selected the one showing the highest expression level for further analysis. Based on its high proline content and experimentally validated role in regulating grain weight, this gene was designated *TaPGW1‐A1* (*Triticum aestivum* Proline‐rich protein regulating Grain Weight 1). To further investigate the transcriptional dynamics of *TaPGW1‐A1* during grain development, qRT‐PCR analysis showed that its transcript level in grains peaked at 3 DPA, then gradually declined (Figure 1b). Consistently, the gene was predominantly expressed in spikes and in developing grains at 3 DPA (Figure 1c), in agreement with the transcriptomic data.

**Figure 1:**
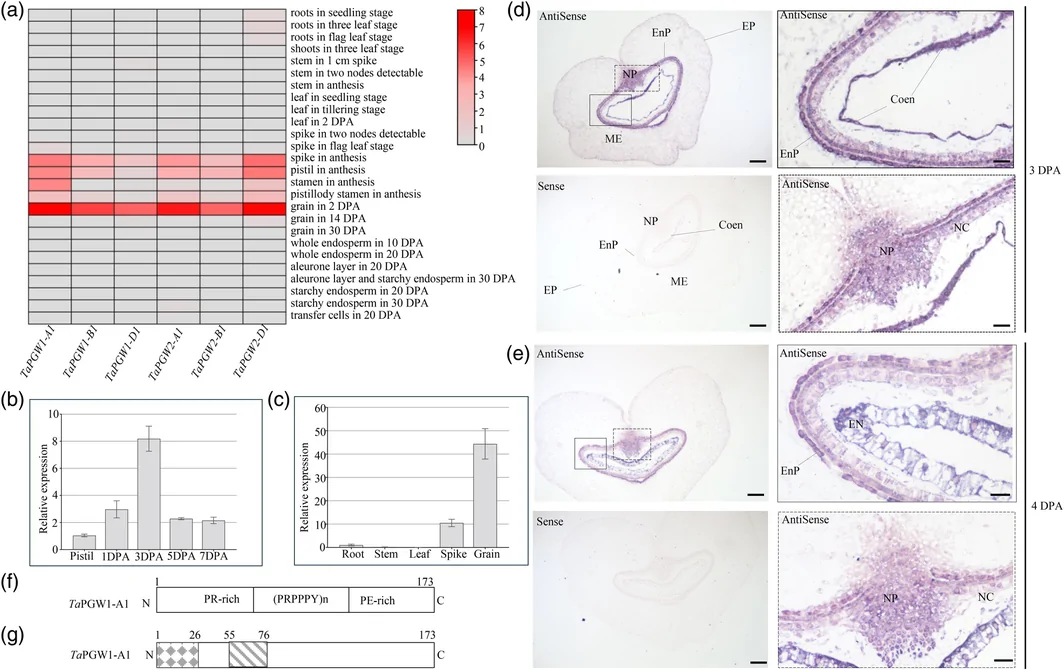
Characterization of *TaPGW1‐A1*. (a) Expression profiles of *TaPGWs* in common wheat. Detailed data are provided in Table S1. (b) Relative *TaPGW1‐A1* expression in pistils/grains at different anthesis stages. DPA—days post anthesis. (c) *TaPGW1‐A1* expression in different tissues at 3 DPA. (d, e) RNA *in situ* hybridization experiments were conducted on 3 DPA and 4 DPA seeds using antisense and sense *TaPGW1‐A1* transcript probes. EP—exocarp; ME—mesocarp; Enp—endocarp; NP—nucellar projection; NC—nucellar; En—endosperm; Coen—coenocyte. Left column scale bars, 200 μm; right column scale bars, 50 μm. (f, g) Schematic representation of *TaPGW1‐A1* motifs. N represents the N terminus, and C represents the C‐terminus. Numbers indicate amino acid positions; the gray region (1–26) denotes the signal peptide and the second gray region (55–76) marks the predicted NLS.

We conducted RNA in situ hybridization (ISH) on cross‐sections of developing grains at 3 DPA (Figure 1d) and 4 DPA (Figure 1e). The results showed that *TaPGW1‐A1* transcripts were detectable in both maternal and filial tissues, including the endocarp, nucellar tissue, nucellar projection, coenocyte, and developing endosperm. These findings provide direct spatial evidence that *TaPGW1‐A1* is expressed in tissues associated with early grain development and support its involvement in the regulation of endosperm cellularization.

We compared the *TaPGW1‐A1* sequence with the genome assembly of Chinese Spring (International Wheat Genome Sequencing Consortium (IWGSC), 2018) and found that the *TaPGW1‐A1* gene is physically located between two high‐confidence genes with continuous numbers, *TraesCS6A02G127600* and *TraesCS6A02G127700*, with a distance of 219 kb on chromosome 6A. However, a low‐confidence gene, annotated as *TraesCS6A02G174100LC*, is present in this large genomic region (Figure S1). *TraesCS6A02G174100LC* is predicted to encode a protein that contains a domain similar to the ethylene‐responsive element binding factor 4 in Arabidopsis (Figure S1). However, TraesCS6A02G174100LC protein is predicted to be translated in the opposite direction. Assuming the *TaPGW1‐A1* transcript was correctly translated from the 5′ to 3′ end, *TraesCS6A02G174100LC* would be erroneously translated from 3′ to 5′.

To determine whether *TaPGW1‐A1* is a functional gene or a pseudogene, we conducted BLAST searches against the Expressed Sequence Tag (EST) database in GenBank to complement our RNA‐seq data. We identified two ESTs, *BM135088* (Figure S1) and *BJ250155* (Figure S1), that matched *TaPGW1‐A1*. The *BM135088* sequence is deposited in the standard 5′ to 3′ orientation, whereas the entry *BJ250155* appears to have been uploaded in the 3′ to 5′ orientation, which is atypical for mRNA sequences that are conventionally presented in 5′ to 3′. Fortunately, the record is clearly labeled as whf9f23 3′, indicating that it represents the 3′ end of the transcript and clarifying its directionality. The combined *BM135088* and *BJ250155* sequences exhibit 100% identity with the *TaPGW1‐A1* transcript sequence (Figure S2a). In order to validate the results, we performed a 3′‐rapid amplification of cDNA ends (RACE) experiment to amplify the full‐length cDNA of *TaPGW1‐A1*.

We further analyzed the *TaPGW1‐A1* sequence across ~30 wheat cultivars included in wheat pangenome sequencing efforts, encompassing more than 10 curated global cultivars (Walkowiak et al., 2020), as well as 17 Chinese cultivars representing the country's breeding history and genomic changes (Jiao et al., 2025). No allelic variation in the *TaPGW1‐A1* coding region resulted in amino acid changes, indicating that the gene has been highly conserved during evolution.

### A unique proline‐rich protein found only in wheat

The *TaPGW1‐A1* gene was predicted to encode a protein of 173 amino acids (a.a.) with a proline content of 35.3% (Figure S2b). The predicted *Ta*PGW1‐A1 protein was found not to have 100% identity with any proteins in the IWGSC RefSeq v1.1 or any other available wheat protein databases. However, it shared 95% identity with the protein XBH77282 from the Chinese Spring T2T genome (Figure S2c) (Liu et al., 2025), and with XP_037455269 (GenBank accession number) (Figure S2d), an extensin‐like protein from the tetraploid wheat *Triticum dicoccoides* genome. These two hypothesized proteins are encoded by *PGW2‐A1*, a duplicated gene within the *TaPGW1‐A1* locus, which differs from the *Ta*PGW1‐A1 protein identified in this study. Therefore, the *TaPGW1‐A1* gene has not been annotated in any sequenced wheat genomes.

To determine whether similar proteins exist in other species or organisms, we conducted BLAST searches against the non‐redundant protein database in GenBank. The results showed that the *Ta*PGW1‐A1 protein has no homologs in any other plant species or organisms, suggesting that *TaPGW1‐A1* is a recently evolved gene. Although it lacks any known canonical domains, the *Ta*PGW1‐A1 protein exhibits a clear modular organization. Its central proline‐rich region contains several distinctive motifs, including a PR‐rich stretch, a PTPPPYT repeat, and a P[E/T/S]‐rich motif (Figure 1f), defining a previously unrecognized subclass of proline‐rich proteins.

Bioinformatic analyses predicted that the *Ta*PGW1‐A1 protein has an N‐terminal hydrophobic signal peptide of 26 a.a., with a cleavage expected between residues 26 and 27, yielding a mature protein of 147 a.a. (Figure 1g; Figure S3a). Hydrophobicity analysis indicated that the protein is largely hydrophilic except for the signal peptide and a small C‐terminal hydrophobic region (Figure S3b). Additionally, a putative nuclear localization signal (NLS) was detected between residues 55 and 76 (Figure 1g; Figure S3c). The absence of natural variation and known conserved domains in the *Ta*PGW1‐A1 protein highlights the need for a transgenic approach to elucidate its functions.

### Increased grain weight in *TaPGW1‐A1* overexpression transgenic wheat

To investigate the biological function of *TaPGW1‐A1*, we generated three independent overexpression (OE) lines in the wheat cultivar “SSD006.” Positive T_0_ transgenic plants were confirmed by PCR using two primer combinations. One primer pair, MAG5423F/MAG9839R, was designed to amplify the *Ubi* + *GUS* fragment in the expression vector (Figure S4a). The other primer pair, WGRA4652F/WGRA4652R, consisting of the *TaPGW1‐A1* upstream primer and a vector‐specific downstream primer, was used to verify the integration of the exogenous transgene (Figure S4b). Subsequently, we performed the same PCR‐based positivity test on the progeny of T_1_ plants; lines showing 100% inheritance (no segregation) in the T_2_ generation were selected as homozygous. We further determined the copy number of *TaPGW1‐A1* and found that all three transgenic lines were homozygous for a single‐copy insertion of the *TaPGW1‐A1* gene (Figure S4c). We then used T_3_ homozygous seeds for all field trials and phenotypic assessments. qRT‐PCR also confirmed that the *TaPGW1‐A1* transcript levels were significantly elevated in these transgenic lines (Figure S4d).

Flowering time was slightly advanced by 3–5 days in transgenic plants, compared with the wild type (WT) in the greenhouse (Figure 2a, b). We evaluated the effects of *TaPGW1‐A1* overexpression on grain yield components in field trials. Compared with WT, the three transgenic lines showed similar effects. Averaged across the three transgenic lines, the transgenic plants (45.84 ± 0.51) increased by 3.2 g per 1000 grains over the WT plants (42.61 ± 0.3) in the first year (Figure 2c, Figure S4e), and the transgenic plants (45.75 ± 1.4) increased by 3.51 g per 1000 grains over the WT plants (42.24 ± 0.42) in the second year (Figure 2d, Figure S4f). Overall, across three transgenic lines and 2 years of field trials, *TaPGW1‐A1* overexpression increased grain weight by 3.4 g per 1000 grains, accounting for an 8% increase.

**Figure 2:**
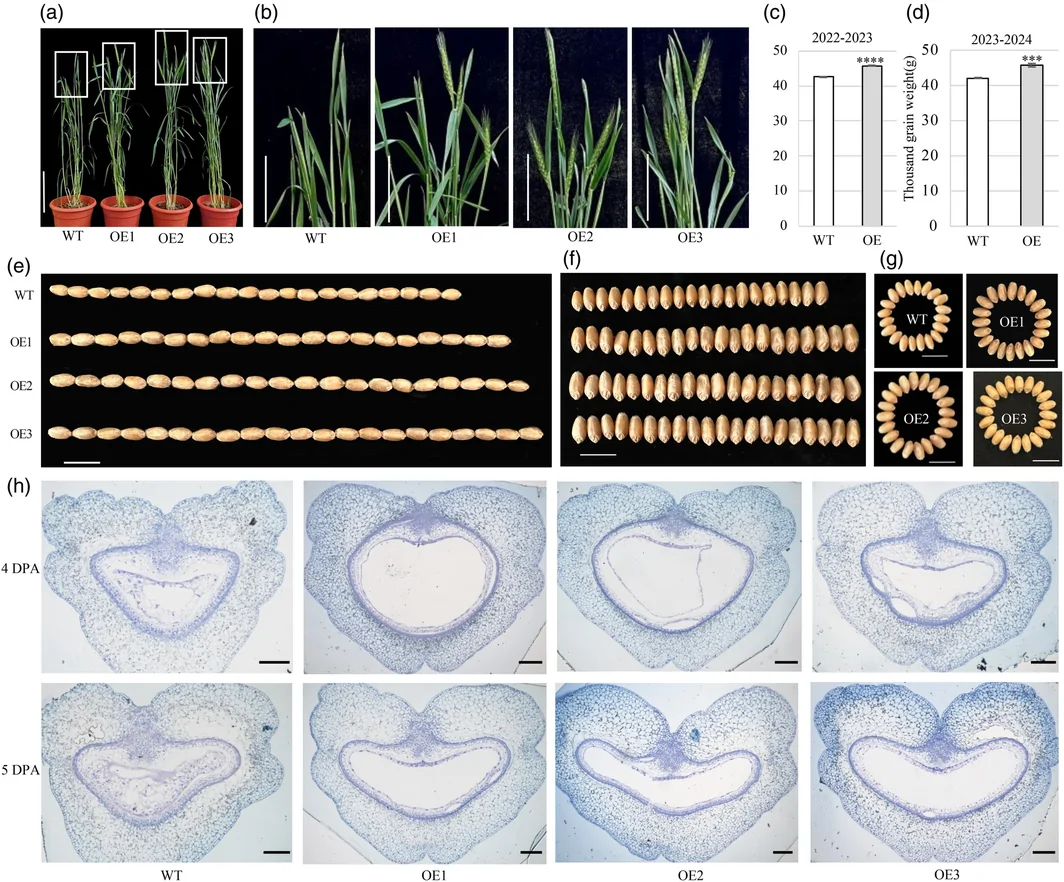
Phenotypes of *TaPGW1‐A1* overexpression transgenic lines. (a) Whole‐plant morphology of WT and *TaPGW1‐A1* OE1‐OE3 lines grown under the same conditions. Scale bar, 20 cm. (b) Magnified partial view of the spike. Scale bar, 10 cm. (c, d) Comparison of 1000‐grain weight between WT and *TaPGW1‐A1* OE plants in 2022–2023 (c) and 2023–2024 (d). The mean 1000‐grain weight from three OE events was statistically analyzed, and the analysis of individual OE events is provided in Figure S4e, f. A *t*‐test was used to determine the significance level between WT and OE, with “***” for *P* < 0.001 and “****” for *P* < 0.0001. The bars indicate standard error. (e–g) Comparison of grain morphology between WT and transgenic plants. Scale bar, 1 cm. (h) Cross‐sections of developing grains from WT and OE1‐OE3 lines at 4 and 5 DPA. Scale bar, 200 μm.

Grain morphology images showed that the significant increase in field‐based 1000‐grain weight was attributable to increased grain length (Figure 2e) and width (Figure 2f, g). Quantitative analyses showed that the increased grain length (Figure S4g) and width (Figure S4h) reached significant levels. By contrast, other agronomic traits, including spikelet number (Figure S4i), spike length (Figure S4j), and grain number per spike (Figure S4k), remained unchanged.

Given that *TaPGW1‐A1* expression peaked at 3 DPA, a critical stage for endosperm cellularization, we examined histological sections of developing grains from both OE and WT lines at 3–6 DPA (Figure 2h, Figure S5a). In sections at 4 and 5 DPA, we observed that endosperm cellularization in the *TaPGW1‐A1* overexpression lines was visibly delayed compared with that in the WT (Figure 2h). Furthermore, at 6 DPA (Figure S5a), when endosperm cellularization was complete and the endosperm cells had filled the entire embryo sac, quantification of endosperm cell nuclei in transverse sections from the overexpression and WT lines revealed that the overexpression lines had more endosperm cell nuclei than the WT (Figure S5b). These results suggest that *TaPGW1‐A1* prolongs the syncytial phase of endosperm development, thereby enlarging final grain size.

### Downstream genes regulated by *TaPGW1‐A1* overexpression in transgenic plants

To elucidate the transcriptional network regulated by *TaPGW1‐A1*, we performed RNA‐seq analysis of 3 DPA grains from *TaPGW1‐A1* overexpression lines and WT plants. PCA analysis based on the top 1000 differentially expressed genes (DEGs) showed overall transcriptomic differences between the WT and OE lines (Figure S6a), indicating that the overexpression of *TaPGW1‐A1* was associated with changes in a global transcriptomic reprogramming. Differential expression analysis identified 175 upregulated (Table S2) and 101 downregulated genes (Table S3). Cell‐cycle‐related genes, including *Cyclin‐H*, *TPR*, and *KIN‐12F*, were concurrently upregulated, suggesting that *TaPGW1‐A1* enhances mitotic activity and coordinates a broad developmental program in cell division. Multiple UPS‐related genes were upregulated, including *UBCc* (E2 conjugase), *RGLG2* (E3 ligase), *SKP1*, and several *F‐box* genes whose products function as substrate‐specific components of the SCF complex. By contrast, a gene encoding a deubiquitinating enzyme (*UCH2*) was downregulated. The transcriptional changes of multiple genes identified by RNA‐seq were validated by qRT‐PCR (Figure S6b). Although the mechanism by which *TaPGW1‐A1* directly or indirectly regulates ubiquitin‐related genes remains unclear, we identified a direct interaction between *Ta*PGW1‐A1 and a ubiquitin receptor.

### Interaction of *Ta*PGW1‐A1 with the ubiquitin receptor *Ta*DSK2a at the cell periphery

To identify molecular partners mediating *Ta*PGW1‐A1 function, we performed a yeast two‐hybrid (Y2H) screen with a cDNA library constructed from wheat spikes collected post‐anthesis. Among the initial interactors detected, *Ta*PGW1‐A1 specifically interacted with the ubiquitin receptor *Ta*DSK2a. DSK2 is a ubiquitin‐binding shuttle protein of the UBA‐UBL family that links ubiquitylated substrates to the proteasome or alternative degradation routes (Elsasser & Finley, 2005). We designed five fragments of *Ta*PGW1‐A1, based on the locations of predicted domains, including three at the N‐terminal (N1, N2, N3) and two at the C‐terminal (C1, C2) (Figure S7a). Each fragment was co‐transformed with *Ta*DSK2a into yeast cells for pairwise interaction assays. Domain‐mapping analysis revealed that the N‐terminal signal peptide (amino acids 1–26) of *Ta*PGW1‐A1 is necessary and sufficient for binding to *Ta*DSK2a (Figure 3a). As controls, another two E3 ubiquitin ligases, *Ta*GW2 and *Ta*RGLG2, did not interact with *Ta*PGW1‐A1 (Figure 3a), demonstrating the specificity of the *Ta*PGW1‐A1–*Ta*DSK2a interaction. The interaction of *Ta*PGW1‐A1 with *Ta*DSK2a was further verified *in planta* by luciferase complementation imaging (LCI, Figure 3b).

**Figure 3:**
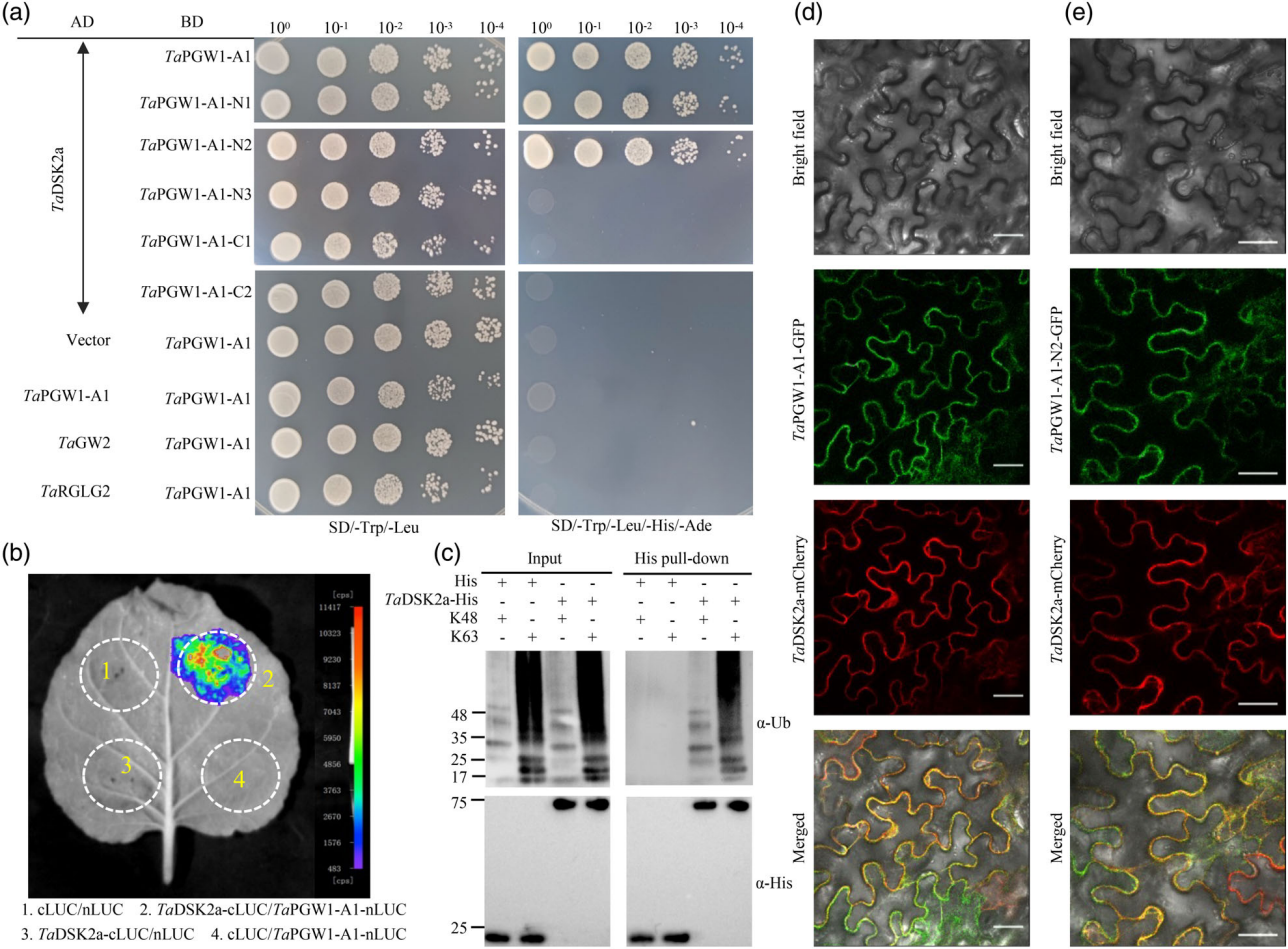
Interaction between *Ta*PGW1‐A1 and *Ta*DSK2a. (a) Interactions of *Ta*PGW1‐A1 with three proteins, *Ta*DSK2a, *Ta*GW2, and *Ta*RGLG2, in Y2H assays. Full‐length and five fragments of *Ta*PGW1‐A1 in the BD vector were tested for interactions with *Ta*DSK2a in the AD vector. *Ta*PGW1‐A1 itself, *Ta*GW2, *Ta*RGLG2, and the empty AD vector were used as controls. Co‐transformed cells were grown on SD media plates lacking two amino acids (−Trp/−Leu), and on SD media plates lacking four amino acids (−Trp/−Leu/−His/−Ade). Five serial dilutions to different folds (10^0^–10^−4^) were inoculated onto the same plate for each protein pair. (b) Interaction of *Ta*PGW1‐A1 and *Ta*DSK2a in a LCI assay. The LCI assay was performed in *N. benthamiana* leaves. nLUC—N‐terminal portion of LUC (the N‐terminal to residue 78); cLUC—C‐terminal portion of LUC (from residue 78 to the C‐terminus). The four combinations were tested on the same leaf. *Ta*PGW1‐A1‐nLUC and *Ta*DSK2a‐cLUC showed interaction signals, but the other three controls did not. (c) Binding of *Ta*DSK2a to K48‐ and K63‐linked polyubiquitin chains through *in vitro* pull‐down assay. *Ta*DSK2a was tagged with His, which was added (+) to different polyubiquitin reactions. His tag was used as a control. (d, e) Interaction of *Ta*PGW1‐A1 with *Ta*DSK2a in *N. benthamiana* leaves. The full‐length (d) and the N2 fragment (e) of *Ta*PGW1‐A1 were fused with a GFP tag, and *Ta*DSK2a was tagged with the mCherry protein. Confocal images show the co‐localization of *Ta*PGW1‐A1‐GFP or *Ta*PGW1‐A1‐N2‐GFP with *Ta*DSK2a‐mCherry at the cell periphery. Scale bar, 20 μm.

We performed *in vitro* ubiquitin‐binding assays using recombinant *Ta*DSK2a‐His. The recombinant *Ta*DSK2a‐His protein bound both K48‐ and K63‐linked polyubiquitin chains (Figure 3c), confirming that *Ta*DSK2a acts as a bona fide ubiquitin receptor capable of engaging substrates destined for proteasomal or non‐proteasomal degradation.

To determine the functional locations of the *Ta*PGW1‐A1 protein, we conducted subcellular localization assays in *Nicotiana benthamiana* epidermal cells using full‐length *Ta*PGW1‐A1, the C‐terminal fragment (*Ta*PGW1‐A1‐C), and the N‐terminal fragment (*Ta*PGW1‐A1‐N2), each fused to a GFP tag (Figure S7b). The GFP tag was used as a control in this study (Figure S7c). *Ta*PGW1‐A1‐C‐GFP was distributed throughout the nucleus and cytoplasm (Figure S7d), whereas full‐length *Ta*PGW1‐A1‐GFP (Figure S7e) and its N‐terminal fragment, *Ta*PGW1‐A1‐N2‐GFP (Figure S7f) showed predominant fluorescence at the cell periphery. Next, *Ta*DSK2a was fused to mCherry. When *Ta*PGW1‐A1‐GFP and *Ta*DSK2a‐mCherry were co‐expressed in the same epidermal cell, the green (GFP) and red (mCherry) signals largely overlapped at the cell periphery (Figure 3d), and a similar pattern was observed upon co‐expression of *Ta*PGW1‐A1‐N2‐GFP and *Ta*DSK2a‐mCherry (Figure 3e). Together, these results indicate that *Ta*PGW1‐A1 and *Ta*DSK2a are spatially associated at the cell periphery and suggest that the N‐terminal signal peptide of *Ta*PGW1‐A1 is required for their co‐localization.

### Essential role of *Ta*DSK2a in grain development and its modulation by the E3 ligase *Ta*RGLG2

We used BSMV‐mediated gene editing to investigate the function of *TaDSK2a* in grain development. Two sgRNA target sites were evaluated in systemically infected leaves by PCR amplification and Sanger sequencing (Figure S8a–c). Editing was detected at the sgRNA‐1 target site in the first exon of *TaDSK2a* (Figure 4a, b), whereas no detectable editing was observed at the sgRNA‐2 target site (Figure S8d). The primary inoculated plants M_1_ displayed chimeric editing patterns, as evidenced by overlapping peaks in the sequencing chromatograms, consistent with the somatic and non‐uniform nature of BSMV‐mediated editing (Figure 4b). Therefore, these plants were used only for mutation induction and preliminary genotyping. Stable edited progeny were then identified in the M_2_ generation, and five independent homozygous M_2_ mutant lines carrying frameshift mutations across the three *TaDSK2a* homeologs were obtained (Figure 4c). These mutations introduced premature stop codons (Figure S8e–g). The phenotypic analyses shown in Figure 4d–h were conducted using these homozygous M_3_ plants. The M_3_ plants used for phenotyping were considered virus‐free, because no viral symptoms were observed and no exogenous *Cas9* sequence was detected. All mutant lines exhibited smaller and less plump grains, with visible reductions in both grain width (Figure 4d) and length (Figure 4e). Quantitative analyses confirmed that the decreases in width (Figure 4f) and length (Figure 4g) were significant, leading to a significant reduction in 1000‐grain weight (Figure 4h), compared with wild‐type plants. Phenotypic analysis of individual edited events is provided in Figure S9. These phenotypes demonstrate that *TaDSK2a* is indispensable for normal grain growth.

**Figure 4:**
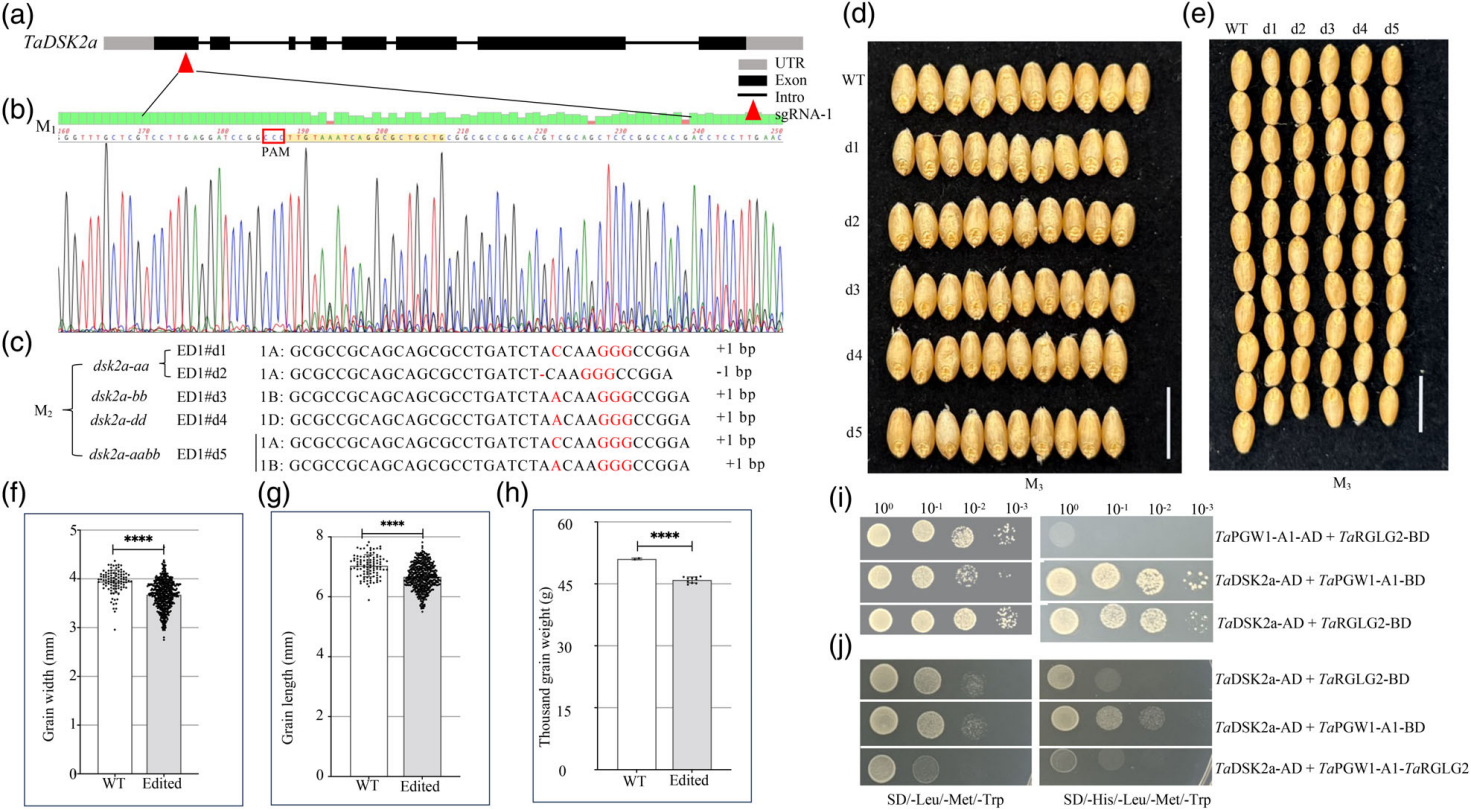
Editing of *Ta*DSK2a and its modulation by *Ta*RGLG2. (a) Diagram of *TaDSK2a* gene structure. The binding site of sgRNA‐1 is indicated with a red arrow. (b) Sequencing chromatogram of edited *TaDSK2a* genes. The sgRNA‐1 binding site is highlighted in yellow, and a red box indicates the PAM motif. Overlapping peaks indicate mixed edited sequences. (c) Individual edited sequences of homoeologous *TaDSK2a* genes. Five edited types include: d1‐d2, homozygous edits in *TaDSK2a‐1A*; d3, a homozygous edit in *TaDSK2a‐1B*; d4, a homozygous edit in *TaDSK2a‐1D*; d5, homozygous edits in *TaDSK2a‐1A* and *TaDSK2a‐1B*. Red GGG denotes PAM; +/− indicates base insertion or deletion. (d, e) Grain morphology of grains from WT and *TaDSK2a‐*edited plants. Scale bar, 1 cm. (f–h) Quantitative analysis of grain width, grain length, and 1000‐grain weight from WT and d1‐d5 genotypes. The mean value from five edited events was statistically analyzed, and the analysis of individual edited events is provided in Figure S9. A *t*‐test was used to determine the significance level between WT and edited plants, with “****” for *P* < 0.0001. The bars indicate standard error. (i) Y2H assays for *Ta*PGW1‐A1, *Ta*RGLG2, and *Ta*DSK2a. (j) Y3H assays for *Ta*PGW1‐A1, *Ta*RGLG2, and *Ta*DSK2a. Transformants were spotted on SD/−Leu/−Met/−Trp and SD/−His/−Leu/−Met/−Trp media. Transformants were serially diluted (10^0^–10^−3^) before spotting onto the selective media.

Although *Ta*PGW1‐A1 did not interact directly with the E3 ligase *Ta*RGLG2 in the previous experiment, Y2H assays confirmed a direct interaction between *Ta*DSK2a and *Ta*RGLG2 (Figure 4i). Yeast tri‐hybrid (Y3H) assays further demonstrated that *Ta*DSK2a could not simultaneously bridge *Ta*PGW1‐A1 and *Ta*RGLG2 into a ternary complex (Figure S10). Instead, co‐expression of *Ta*RGLG2 inhibited the *Ta*PGW1‐A1–*Ta*DSK2a interaction. Y3H assays showed that *Ta*RGLG2 reduced the interaction signal between *Ta*PGW1‐A1 and *Ta*DSK2a (Figure 4j). This suggests that *Ta*RGLG2 may interfere with the *Ta*PGW1‐A1–*Ta*DSK2a interaction.

## DISCUSSION

Our findings suggest that important regulators can emerge from genomic regions once regarded as non‐genic or functionally inert. Although it is difficult to demonstrate that an ancestral sequence was truly without function, studies of plant de novo genes show that previously unannotated intergenic sequences can acquire transcription and biological roles. For example, in Arabidopsis, *TWT1* has been proposed to originate from a non‐genic sequence and to affect microtubule organization and helicoidal growth (Marsch‐Martínez et al., 2022). In rose, the de novo‐originated gene *SCREP* contributes to scent diversification, and a transposable element insertion in its promoter enhances its expression (Li et al., 2025). Consistent with this broader pattern, *TaPGW1‐A1* appears to represent a similar innovation in wheat. *TaPGW1‐A1* is embedded within a 219‐kb intergenic region on chromosome 6A and is found exclusively in wheat, suggesting that it originated during wheat genome evolution. *TaPGW1‐A1* was duplicated into two copies in each of the homoeologous A, B, and D genomes of hexaploid wheat (Figure S11, Table S4). Sequence comparisons indicate that *TaPGW2‐A1*/*‐B1*/*‐D1* and *TaPGW1‐B1*/*‐D1* share high sequence similarity with *TaPGW1‐A1* and are located in duplicated genomic regions, suggesting that they arose from recent gene duplication events during wheat polyploidization. However, whether they represent functional duplications or pseudogenes requires further investigation.

To further investigate whether this gene is unique to the *Triticeae*, we analyzed its homologs in six representative *Triticeae* genomes (after confirming its absence in non‐*Triticeae* species such as rice and maize). These include two AA genomes (AA in *T. urartu* and A^m^A^m^ in *Triticum monococcum*), one DD genome (*Aegilops tauschii*), one SS genome (the putative ancestor of the BB genome), and two AABB genomes (durum and wild emmer wheat). The genomic coordinates of these homologs are summarized in Table S4. Notably, in the wild emmer genome, we identified an extra *PGW* gene copy on chromosome 6B (chr6B:152732580–152 732 751). Because this sequence is unusually short and lacks features typical of functional *TaPGW* genes, it may represent either a pseudogene or an annotation artifact. By contrast, no *TaPGW1‐A1* homolog was identified in the barley genome, a closely related temperate cereal, suggesting that *TaPGW1‐A1* originated after the divergence of the wheat and barley lineages approximately 11–12 million years ago.

*Ta*PGW1‐A1 is highly enriched in proline (35.3%), consistent with the general features of proline‐rich proteins, yet it diverges from classical proline‐rich proteins in both sequence and function. The *Ta*PGW1‐A1 protein contains unique proline‐rich motifs but lacks canonical domains. Proline‐rich proteins have traditionally been regarded as structural components of the cell wall (Cassab, 1998; Showalter, 1993). However, members of this family can undergo rapid diversification in both sequence and expression pattern, and some have acquired functions beyond their canonical structural roles (Dvořáková et al., 2007). Consistent with this evolutionary diversification, *TaPGW1‐A1* appears to have acquired a distinct regulatory role in wheat grain development.

Unlike conventional proline‐rich proteins that primarily serve as structural components of the cell wall (Showalter, 1993; Cassab, 1998), *Ta*PGW1‐A1 exhibits distinctive molecular and developmental characteristics. Its expression peaks at 3 DPA, coinciding with the transition from syncytial to cellularized endosperm. Multi‐environment field trials further demonstrate that this specialization translates into consistent increases in grain weight when *TaPGW1‐A1* is overexpressed. Collectively, these attributes indicate that *Ta*PGW1‐A1 represents a functionally diverged member of proline‐rich proteins that has acquired specialized regulatory roles in wheat grain development.

This functional expansion of a proline‐rich protein‐related sequence into a signaling regulator reflects a broader evolutionary trend in plants. *TaPGW1‐A1* may therefore represent an example of neofunctionalization, in which a lineage‐specific proline‐rich protein has acquired regulatory functions in cellular processes distinct from its ancestral structural role. One limitation of this study is the use of the constitutive rice *Actin1* promoter to generate *TaPGW1‐A1* overexpression lines. Because this promoter drives transgene expression in multiple tissues, it may not fully recapitulate the native spatiotemporal expression pattern of *TaPGW1‐A1* and could potentially introduce minor pleiotropic effects beyond grain development. Although no obvious alterations in vegetative growth or reproductive architecture were observed, future studies using tissue‐specific promoters and detailed analyses of additional organs will be important to determine whether *TaPGW1‐A1* has functions outside grain development.

In contrast to the apparent wheat‐specific origin of *TaPGW1‐A1*, many genes controlling grain or seed size in model plants such as rice and Arabidopsis are evolutionarily more conserved. For example, the rice *GW2* encodes a RING‐type E3 ubiquitin ligase that negatively regulates grain width and grain weight (Song et al., 2007), and this regulatory role has been shown to be broadly conserved in cereals, including wheat (Jaiswal et al., 2015). By comparison, *TaPGW1‐A1* appears to represent a lineage‐specific regulator rather than a direct counterpart of these conserved grain‐size genes. In Arabidopsis, key regulators of endosperm development and cellularization, such as *FIS2* and *MEA*, function primarily through epigenetic mechanisms associated with Polycomb group complexes (Wang et al., 2006), which differs from the ubiquitin–proteasome–related pathway associated with *TaPGW1‐A1*. These findings suggest that although ubiquitin‐mediated protein turnover is a conserved principle in seed development (Lv et al., 2022), distinct regulatory components may have evolved in different plant lineages (Hua & Yu, 2019). Notably, the proline‐enriched nature of *Ta*PGW1‐A1 and its interaction with the ubiquitin receptor have not, to our knowledge, been reported for analogous regulators in rice or Arabidopsis, raising the possibility that *TaPGW1‐A1* represents a previously unrecognized regulatory component in wheat grain development.

The key molecular connection underlying *Ta*PGW1‐A1 function is its direct interaction with the ubiquitin receptor *Ta*DSK2a through its N‐terminal signal peptide. The markedly reduced grain size observed in *Ta*DSK2a knockout plants underscores the importance of this receptor in the regulatory circuit. The finding that the E3 ligase *Ta*RGLG2 affects the *Ta*PGW1‐A1–*Ta*DSK2a interaction suggests an additional regulatory layer that helps maintain proteostasis and developmental balance. Based on our data, we propose a mechanistic model (Figure S12), in which *Ta*PGW1‐A1 co‐localizes with *Ta*DSK2a at the cell periphery during early grain development. Given its ability to recognize K48‐ and K63‐linked polyubiquitin chains, *Ta*DSK2a may participate in selective substrate turnover, thereby affecting cell‐cycle progression and the timing of endosperm cellularization. Our interaction assays further suggest that *Ta*RGLG2 also associates with *Ta*DSK2a and may modulate the *Ta*PGW1‐A1–*Ta*DSK2a interaction, although the precise relationship among these three proteins requires further investigation. Collectively, the *Ta*PGW1‐A1–*Ta*DSK2a–*Ta*RGLG2 module may represent a regulatory link between developmental phase transitions and ubiquitin‐mediated protein degradation, providing a useful framework for understanding endosperm development and a potential genetic entry point for improving wheat yield potential.

## MATERIALS AND METHODS

### Plant materials and growth conditions

The recombinant inbred line SSD006 (derived from Nanda 2419 × Wangshuibai) was used for transformation. Wangshuibai served as the donor for cloning *TaPGW1‐A1*. Three independent *TaPGW1‐A1* overexpression lines (OE1‐OE3) and the wild type (WT, SSD006) were used to evaluate grain traits (1000‐grain weight, grain length, width), spike length, spikelet number, and grains per spike. Field trials were conducted at the Baima and Pailou Experimental Stations of Nanjing Agricultural University, Jiangsu, China (32°04′ N, 118°48′ E). Only grain weight was evaluated using materials collected from two locations, whereas all other traits were analyzed using samples from the Pailou experimental field, samples for RNA‐seq were collected at Baima. The BSMV editing receptor line NMAS001m (laboratory line carrying *Cas9*) and homozygous *TaDSK2a* mutants (d1‐d5), and the WT were grown in the above‐mentioned Pailou experimental field for phenotypic evaluation. Trials followed a randomized block design with two replicates. Each lot is 0.3 × 1.5 m in area, where 20 seeds were evenly sown. Field management followed local standard practices.

For gene‐expression assays, plants were grown in controlled chambers at 25°C/18°C (day/night; long days, 18 h light/6 h dark). Ovaries or grains were collected at pistil, 1, 3, 5, and 7 DPA for time‐course analyses. Tissues (root, stem, leaf, spike, and grain at 3 DPA) were harvested for expression profiling. Samples for resin sectioning were collected under the growth conditions described above. Specifically, wild‐type and *TaPGW1‐A1* overexpression (OE1‐OE3) grains at 3, 4, 5, and 6 DPA were collected for resin sectioning. *TaPGW1‐A1* overexpression T_3_ homozygous lines (OE1‐OE3) were used for all field trials, grain trait measurements, RNA‐seq, and histological analyses.

### RNA extraction and gene‐expression analysis

Total RNA was extracted using TRIzol (Invitrogen, Thermo Fisher Scientific, Carlsbad, CA, USA) according to the manufacturer's protocol. First‐strand cDNA was synthesized using EasyScript® (TransGen Biotech Co., Ltd., Beijing, China) First‐Strand cDNA Synthesis SuperMix (TransGen Biotech). Quantitative real‐time PCR (qRT‐PCR) used TransStart® Green qPCR SuperMix (TransGen Biotech). *TaTubulin* served as the endogenous reference, and relative expression was calculated by the 2^ ‐ΔΔCT (Livak & Schmittgen, 2001) method. Each assay included three biological and three technical replicates.

### Vector construction and genetic transformation

The full‐length coding sequence (CDS) of *TaPGW1‐A1* was amplified from Wangshuibai cDNA using primers MAG4504F/MAG4504R containing a PstI restriction site. The PCR product was ligated into a PstI‐digested pUC19‐based intermediate vector carrying the *Actin1* promoter from rice (*OsActin1*, GenBank accession S44221, 1.3 kb upstream region) and the nopaline synthase (Nos) terminator from *Agrobacterium tumefaciens* using T4 DNA ligase (M0202S, NEB), generating the *Actin1*::*TaPGW1‐A1*::*Ter* expression cassette. The resulting *Actin1*::*TaPGW1‐A1*::*Ter* expression cassette was then excised with HindIII and ligated into the HindIII‐digested pUCBS vector using T4 DNA ligase (M0202S, NEB), generating the final biolistic transformation vector (Figure S13). pUCBS is a 14 066‐bp vector backbone maintained in our laboratory for stable transformation, containing a *GUS* marker gene‐expression cassette and an *NPTII* selectable marker expression cassette.

Immature embryos (~14 DPA) were used for genetic transformation. Seeds were surface‐sterilized with 70% ethanol for 5 min, rinsed with sterile water, treated with 2% sodium hypochlorite for 7–8 min, and rinsed again. Immature embryos were excised and cultured scutellum side up on MS induction medium (4.4 g L^−1^ MS basal salts, 30 g L^−1^ sucrose, 75 mg L^−1^ aspartic acid, 150 mg L^−1^ glutamine, 1 g L^−1^ MES, 5 g L^−1^ agar, and 1 mg L^−1^ 2,4‐D, with the pH adjusted to 5.8) at 25°C in the dark for 3 days. The embryos were then transferred to osmotic medium (4.4 g L^−1^ MS basal salts, 30 g L^−1^ sucrose, 0.2 mol L^−1^mannitol, 0.2 mol L^−1^ sorbitol, 1 g L^−1^ MES, 5 g L^−1^ agar, and 1 mg L^−1^ 2,4‐D, with the pH adjusted to 5.8) for 4–6 h before biolistic transformation. Thirty minutes before bombardment, 5 μg plasmid DNA was coated onto 1 mg gold particles (0.6 μm, Bio‐Rad) (Jones & Sparks, 2009).

A PDS‐1000/He particle delivery system for genetic transformation, as well as rupture disks, stopping screens, and macrocarriers, were purchased from Bio‐Rad (USA). The stopping screens, rupture disks, and macrocarriers were sterilized by soaking in 70% ethanol and then air‐dried on sterile filter paper in a laminar‐flow hood. A 10 μL aliquot of plasmid‐coated gold particles was loaded onto the center of the macrocarrier. After the ethanol had completely evaporated, the macrocarrier was placed into the cartridge with the coated side facing upward, followed by sequential assembly of the rupture disk, stopping screen, and Petri dish. The Petri dish was placed on the third shelf, and the macrocarrier was placed on the first shelf. The chamber was evacuated to 28 inch Hg, and each dish was bombarded once. After bombardment, the calli were cultured on the original osmotic medium in the dark for 16–18 h and then transferred to MS induction medium for further culture at 25°C in the dark.

Immature embryos were initially cultured on induction medium without selection pressure for 30–40 days. This period allowed transformed cells to proliferate and form calli. The resulting calli were then transferred to a selection medium containing 30 mg/L G418 and maintained under continuous selection until shoot regeneration. This was determined based on preliminary optimization experiments and was sufficient to effectively inhibit the growth of non‐transformed cells while allowing the recovery of transformed events. The calli were cultured on the MS selection medium supplemented with 30 mg L^−1^G418 (4.4 g L^−1^ MS basal salts, 30 g L^−1^ sucrose, 1 g L^−1^ MES, 5 g L^−1^ agar, 1 mg L^−1^ KT, and 30 mg L^−1^ G418, with the pH adjusted to 5.8) for shoot induction at 25°C under a 12‐h photoperiod. The selected regenerated plantlets were then transferred to MS rooting medium (40 g L^−1^ MS, 1 g L^−1^ MES, and 3.5 g L^−1^ agar, with the pH adjusted to 5.8) and cultured at 25°C under a 12 h photoperiod. After rooting, the plantlets were transferred to pots for subsequent positive identification.

Genomic DNA was extracted by the SDS method (Ma & Sorrells, 1995) and transgenics were confirmed by PCR‐based identification of transgenic plants using two primer pairs: MAG5423F/MAG9839R, which specifically amplify the *Ubi + GUS* fragment in the expression vector; and WGRA4652F/WGRA4652R, which were used to confirm the integration of the exogenous transgene. Products were resolved on 1.5% agarose gels.

Quantitative real‐time PCR (qRT‐PCR) was performed to determine the transgene copy number (Zhang et al., 2015). Genomic DNA extracted from leaves of WT and transgenic plants was used as the template. The wheat single‐copy gene *e‐LCY3A* (*Lycopene ε‐cyclase 3A*) served as the endogenous reference, and the *TaPGW1‐A1* copy number was determined with the specific primer pair MAG7107. qRT‐PCR used TransStart® Green qPCR SuperMix (TransGen Biotech). Each sample was analyzed with three technical replicates. Relative quantification was calculated using the 2^ ‐ΔΔCT (Livak & Schmittgen, 2001) method. Plants with relative copy number values approaching 2.0 were considered homozygous lines carrying a single‐copy insertion.

### BSMV‐sgRNA gene editing of *TaDSK2a*

Two sgRNAs targeting exon 1 of *TaDSK2a* (homeologs 1A/1B/1D) were designed using the E‐CRISP web tool. The BSMV ND18 α/β/γ vectors (Scofield Lab, Kansas State University) were used. Two gRNA cassettes were PCR‐amplified from pBUE411 and inserted by homologous recombination into the γ vector, which had been linearized with NotI and PacI. Plasmids were prepared by alkaline lysis; BSMV α and γ (with insert) were linearized with MluI and BSMV β with SpeI. In vitro transcription/capping was performed using the HiScribe® T7 kit with Cap Analog (NEB); 20 μL reactions were incubated at 37°C for 2 h and analyzed by 2% agarose electrophoresis.

BSMV‐sgRNA transcripts were mechanically inoculated onto the second leaf of two‐leaf‐stage wheat plants, and the inoculation procedure was performed essentially as previously described (Jian et al., 2017). Two weeks after inoculation, the newly emerged leaves were collected for DNA extraction and genotyping to assess whether editing had occurred. Locus‐specific primers for *TaDSK2a‐1A/1B/1D* amplified targets for Sanger sequencing; superimposed chromatogram peaks at the target sites indicated successful editing. The plants regenerated through tissue culture and carrying CRISPR/Cas9‐induced mutations are now designated as the M_1_ generation, their selfed progeny as M_2_, and the next generation as M_3_. Because BSMV‐mediated editing in the M_1_ generation is somatic, the resulting M_1_ plants frequently exhibited chimeric editing patterns. Therefore, M_1_ plants were used only for mutation detection and transmission analysis. Stable edited progenies were subsequently identified in the M_2_ generation by PCR amplification and Sanger sequencing, and only homozygous M_3_ plants were used for phenotypic analysis. These M_3_ plants were considered virus‐free because they showed no viral symptoms, and no exogenous *Cas9* sequence was detected. Therefore, the phenotypic analyses were performed on heritably edited plants without evidence of persistent viral infection or continued presence of the editing reagent.

### Agronomic trait measurements

Mature seeds from Pailou and Baima field trials were used for grain trait measurements. Grain length and width were measured using Intelligent Seed Analyzer; 1000‐grain weight was determined with an analytical balance (Sartorius). For WT and each OE line, grain dimensions were measured using a seed analyzer (TPKZ‐3, TOP Cloud‐Agri) with 150 grains per line, and 1000‐grain weight was determined using four biological replicates. Spike length, spikelet number, and grains per spike were recorded from 10 plants per line before harvest. *TaDSK2a* mutant plants were grown in a controlled chamber; grain length/width (100 grains per line) and 1000‐grain weight (two replicates) were measured as above.

### RNA‐seq analysis

Sixty‐nine RNA‐seq samples, mainly from the wheat variety Chinese Spring and representing diverse organs and developmental stages, were obtained from URGI. Clean reads were aligned to the IWGSC reference genome (IWGSC RefSeq v1.1 (International Wheat Genome Sequencing Consortium (IWGSC), 2018)) using STAR (Dobin et al., 2013). A comprehensive annotation file was created by merging the official IWGSC RefSeq v1.1 annotation with genes assembled *de novo* from the data using StringTie (Pertea et al., 2015). Read counts were then generated from uniquely mapped reads using featureCounts (Liao et al., 2014) with this combined annotation, a strategy employed to minimize artifacts in the hexaploid wheat genome. Gene‐expression levels were quantified as Transcripts Per Million (TPM).

Based on their developmental profiles, the samples were categorized into two groups: seven samples from early spike or grain stages (Group I) and the remaining samples (Group II). Six *TaPGW* genes were selected for further study based on the following criteria: (1) an average TPM in Group I greater than 9, indicating extremely high expression; and (2) an average TPM in Group II less than 0.1, indicating nearly undetectable expression. Heatmaps of expression data for six *TaPGW* genes across wheat development were generated via TBtools‐II (Chen et al., 2023).

Grains from WT and *TaPGW1‐A1* overexpression lines (OE1‐OE3) were collected at 3 DPA (two biological replicates per genotype; eight libraries total). The RNA‐seq data were analyzed using the same pipeline as the public data. *TaPGW1‐A1* expression was verified by qRT‐PCR before sequencing. Libraries were sequenced on Illumina X10 (Beijing Novo Technology). Sample concordance was evaluated using plotMDS in R. Differentially expressed genes (DEGs) were identified using edgeR (FDR <0.05; fold change >1.5). Functional annotations were curated from UniProt and the literature.

### *Ta*PGW1‐A1 protein characterization

Protein domains were queried in Pfam. Signal peptide, NLS, and hydrophobicity were predicted using SignalP 5.0, NLStradamus, and ProtScale, respectively.

### RNA *in situ* hybridization

RNA *in situ* hybridization was conducted on developing seeds collected from Pailou experimental field at 3 and 4 DPA. The samples were immersed in FAA solution (50% ethanol, 5% glacial acetic acid, and 4% formaldehyde) at 4°C for 24 hours. After fixation, samples were dehydrated through a graded butanol series and embedded in paraffin. Sections of 7 μm were cut, deparaffinized, rehydrated, and digested with 1 μg/mL proteinase K at 37°C for 30 min, followed by three washes with 1 × PBS. Sections were pre‐equilibrated in DIG‐EASY‐HYB buffer at 37°C for 30 min, then hybridized with digoxigenin‐labeled probes (10–50 pg/mL) at 42°C for 18 h. After hybridization, sections were washed twice with 2 × SSC at room temperature and twice with 0.5 × SSC at 65°C. They were then incubated sequentially with washing buffer, blocking solution, and anti‐digoxigenin‐AP solution (DIG Nucleic Acid Detection Kit, NBT/BCIP; Roche). Following two washes with washing buffer, the sections were equilibrated in detection buffer and incubated with substrate solution in the dark until a purple signal developed. The reaction was stopped with TE buffer. Probes were prepared according to the instructions of the DIG RNA Labeling Kit (SP6/T7; Roche). The sequences of sense and antisense RNA probes are provided in Table S5. Three replicates were performed.

### Histological analysis

Grains from WT and *TaPGW1‐A1* overexpression lines were collected at 3–6 DPA. Grains were fixed in glutaraldehyde solution and vacuum‐infiltrated at 0.02–0.03 MPa for 30 min, then stored at 4°C. Resin embedding followed the EMbed‐812 kit (Electron Microscopy Sciences): graded ethanol dehydration (30%, 70%, 100%), graded propylene oxide exchange (30%, 70%, 100%), infiltration in 25%, 50%, and 100% resin, and polymerization at 65°C for 10 h. Sections (3 μm) were cut on a Leica EM UC7 microtome, stained with toluidine blue (2–5 min), rinsed, dried, and imaged on a Zeiss microscope. The experiment was repeated twice for transgenic lines and WT. Endosperm cell nuclei counts were quantified from resin sections using ImageJ.

### Y2H and Y3H assays

The *TaPGW1‐A1* CDS was cloned into the BD vector pGBKT7 to generate *Ta*PGW1‐A1‐BD. A spike cDNA library (flowering stage, WSB) was constructed per Clontech protocols. Y2H screening and validations used the Matchmaker™ system (Clontech). Self‐activation of *Ta*PGW1‐A1‐BD was tested in the Gold strain. For pairwise interactions, *TaPGW1‐A1*, *TaDSK2a* (*TraesCS1B02G217000*), *TaGW2* (*TraesCS3B02G449200*), and *TaRGLG2* (*TraesCS6A01G189300*) CDSs were cloned into the AD vector pGADT7 to produce AD fusions (*Ta*PGW1‐A1‐AD, *Ta*DSK2a‐AD, *Ta*GW2‐AD, *Ta*RGLG2‐AD). *Ta*PGW1‐A1 truncations for mapping were cloned into pGBKT7: N‐terminal (N1, signal peptide + NLS; N2, signal peptide only; N3, partial signal peptide + NLS) and C‐terminal (C1, lacking signal peptide and NLS; C2, partial NLS). Co‐transformants were serially diluted (10^0^–10^−4^) and spotted on SD/−Trp/−Leu and SD/−Trp/−Leu/‐His/−Ade.

For Y3H assays, *TaPGW1‐A1*, *TaDSK2a*, and *TaRGLG2* CDSs were cloned into pBridge to generate RGLG2‐BD, RGLG2‐DSK2a, PGW1‐A1‐BD, and PGW1‐A1‐RGLG2 constructs. Yeast co‐transformants were plated on SD/−Leu/−Met/−Trp and SD/‐His/−Leu/−Met/−Trp after serial dilution (10^0^–10^−3^) and incubated at 30°C for 3–5 days.

### Luciferase complementation imaging (LCI)

*TaPGW1‐A1* and *TaDSK2a* CDSs were cloned into pCAMBIA1300‐nLUC and pCAMBIA1300‐cLUC (*Ta*PGW1‐A1‐nLUC, *Ta*DSK2a‐cLUC), transformed into *Agrobacterium tumefaciens* GV3101, and co‐infiltrated into *N. benthamiana* leaves (Liang & Li, 2022). After 48 h under light, leaves were sprayed with D‐luciferin potassium salt, dark‐adapted for ~5 min, and imaged on a Berthold LB 985 system.

### Subcellular localization and co‐localization

Full‐length *TaPGW1‐A1* and two truncations (*Ta*PGW1‐A1‐C lacking the signal peptide; *Ta*PGW1‐A1‐N2 containing only the signal peptide) were fused to GFP in pCAMBIA2300‐GFP. *TaDSK2a* was fused to mCherry in pCAMBIA1300‐mCherry. Constructs were transformed into GV3101 and infiltrated into *N. benthamiana* leaves. After 48 h, fluorescence was imaged on a Leica SP8 confocal microscope (GFP excitation 488 nm; mCherry excitation 550 nm).

### Ubiquitin‐chain binding assays

*TaDSK2a* CDS was cloned into pET32a to express *Ta*DSK2a‐His in *Escherichia coli* BL21 (DE3). Expression was induced with 0.5 mM IPTG at 16°C for 24 h, and protein was purified by Ni‐affinity chromatography (Smart‐Lifesciences). Purified *Ta*DSK2a‐His was incubated for 4 h at 4°C with Lys‐48‐linked (UBPBio D1501) or Lys‐63‐linked (UBPBio D2600) polyubiquitin in buffer A (50 mM Tris–HCl, pH 8.0; 300 mM NaCl; 20 mM imidazole; 1 mM β‐mercaptoethanol; 1 mM PMSF). Bound proteins were eluted by heating in SDS sample buffer, separated by SDS‐PAGE, and immunoblotted with anti‐ubiquitin (1:1000, Beyotime AF1705) and anti‐His (1:2000, EASYBIO BE2017). The membranes were then incubated with an HRP‐conjugated goat anti‐rabbit & mouse IgG secondary antibody (1:10000, Abmart M21003).

All primers used in this study are listed in Table S5. All databases used for sequence acquisition and bioinformatic analyses are summarized in Table S6.

## AUTHOR CONTRIBUTIONS

HJ and ZM conceived and supervised the project. LG, SD, DH, and ZG performed the experiments. YY conducted the bioinformatic analyses. SL and LM contributed to phenotypic evaluations. QX and ZM provided critical suggestions and discussion. HJ and LG wrote the manuscript. All authors reviewed and approved the final version of the manuscript.

## CONFLICT OF INTEREST

The authors declare no competing interests.

## Supporting information


**Figure S1.** Sequences from public data used to verify and reconstruct the *TaPGW1‐A1* gene.
**Figure S2**. Sequence comparison of *TaPGW1‐A1*, XBH77282, and XP_037455269.
**Figure S3**. Characterization of *Ta*PGW1‐A1 protein.
**Figure S4**. Genotype and phenotype analyses of *TaPGW1‐A1* overexpression plants.
**Figure S5**. Endosperm cellularization in *TaPGW1‐A1* overexpression transgenic wheat.
**Figure S6**. RNA‐seq analyses of *TaPGW1‐A1* overexpression lines.
**Figure S7**. *Ta*PGW1‐A1 fragments and subcellular localization.
**Figure S8**. BSMV‐mediated editing of *TaDSK2a*.
**Figure S9**. Phenotypic analysis of edited *TaDSK2a* plants.
**Figure S10**. Y3H assays for *Ta*PGW1‐A1, *Ta*RGLG2, and *Ta*DSK2a.
**Figure S11**. Sequences and physical locations of *TaPGWs*.
**Figure S12**. Proposed working model for the *Ta*PGW1‐A1–*Ta*DSK2a–*Ta*RGLG2 regulatory module.
**Figure S13**. Schematic representation of the overexpression vector for *TaPGW1‐A1*.

**Table S1.** RNA‐seq data used in this study and TPM values for six *TaPGW* genes.

**Table S2.** Upregulated genes identified by RNA‐seq analysis.

**Table S3.** Downregulated genes identified by RNA‐seq analysis.

**Table S4.** Summary of *TaPGW1‐A1* homologous gene copies in *Triticeae*.

**Table S5.** Primers used in this study.

**Table S6.** Databases used in this study.

## Data Availability

All data supporting the findings of this study are available within the paper and its Supplementary Information files. The raw RNA‐seq data have been deposited in the Genome Sequence Archive (GSA) at the China National Center for Bioinformation (CNCB‐NGDC) under accession number CRA033006.
