# Supplementary material for: A wheat‐specific proline‐rich protein regulates grain weight through a ubiquitin‐mediated delay of endosperm cellularization
Source: Plant J. 2026 Oct 10;128(1):e71148. doi: 10.1111/tpj.71148 (PMC13653226; doi:10.1111/tpj.71148)
Supplement: Supplementary file 1 [file TPJ-128-0-s002.docx]

**A wheat-specific proline-rich protein regulates grain weight through a ubiquitin-mediated delay of endosperm cellularization**

>**a,** TraesCS6A02G174100LC

ATGGCGGCTCCTCGTAGTCGGCCGCGTCGGCGGCAGCCTGTACGCCAGCGGAGTCGGCTTCCGCGCCGTGGTCGAGGAAGGGAGATGGCATCGGGGCCGGGGCGTATGGGGGTGGCGTGGGCTCAGGAGGGGACGGAGGGGACGGAGTGCACGGCGGCGGCGTTGGCGTGGGCTCAGGCTCAGGCTCAGGAGGGGACGGAGTGGGTTCAGGCTCAGGAGGGGACGGAGTGGGCGGGGGCGAGGGCGAGGGCGAGGGCGCGGCGGATGCGGACGCGGCCTGGGAGGCTTCCTGTTGCTGTGGGAGGTGGTGAGCGGCGTCGGGGCGGGGTCGTAGCCGCCGCTGTCCGCCTGCTGGGTCTCCAAGGCCAGGCTCAGGAGCAGCAGGGCCACCAGCAGAGCGGCTTTGCCACCCGCCATTCTCGCACTCCCTCACCGCCTCACGCACTGCAAAGTTCTTGGCTCGATCGGTGTGGTTCTGGGAAGAAGAGTGCACCCATACCACTGCTTATATACACGGAGGGAGGAGTCGCGTCCTTTGCTTGTGCGCGCAAGCGATGGAGCTGGCGCTATGCGGGGGAGGTTGGACACTCTGGAGTTACCCGAACTGGGCAAACCCTCATGAACGGCGTTGTCTGCCGAAACAAGCTCCGACCCGCCTGCCGTAGCTCCGACCCGCCCCGGCTCCGTCCGCCGCCGCCCCGCAGCTCTGCCCCGCCCGGCTCCGTCTGCCACCGCCCCGGCCGCACGCTGCCGCCGCTCCACCGCTCTCACCGCTCTCCAATAGTGCCGAAGAAGACGTCGAAGGGCAAGTCGGGCTTCTTCGGCGTGAGGCAGAAGCCCTCCGGTAAATCCGGAGTGGAGTTCTCCGACGCCGGGAGGCGTTGGTGGATTGGCACATACCCCGCCCACGAGGCCGCGCGTGCCTACGACGTGGCACTGTGGCGTGCCGAGAGGCCTCGGTAG

>**b,** TraesCS6A02G174100LC

MAAPRSRPRRRQPVRQRSRLPRRGRGREMASGPGRMGVAWAQEGTEGTECTAAALAWAQAQAQEGTEWVQAQEGTEWAGARARARARRMRTRPGRLPVAVGGGERRRGGVVAAAVRLLGLQGQAQEQQGHQQSGFATRHSRTPSPPHALQSSWLDRCGSGKKSAPIPLLIYTEGGVASFACARKRWSWRYAGEVGHSGVTRTGQTLMNGVVCRNKLRPACRSSDPPRLRPPPPRSSAPPGSVCHRPGRTLPPLHRSHRSPIVPKKTSKGKSGFFGVRQKPSGKSGVEFSDAGRRWWIGTYPAHEAARAYDVALWRAERPR*

>**c,** Comparison of protein sequences

TraesCS6A02G174100LC GVRQKPSGKSGVEFSDAGR--RWWIGTYP-AHEAARAYDVA

GVR++P G+	E D G+ R W+GT+ A EAARAYD A

*At*ERF (NP_188139.1)	GVRKRPWGRYAAEIRDPGKKTRVWLGTFDTAEEAARAYDTA

>**d,** BM135088, Triticum aestivum cDNA clone WHE0459_G04_G04, mRNA sequence

AGGGAACTTTGCAGTGCGTGAGGCGGTGAGGGAGTGCGAGAATGGCGGGTGGCAAAGCCGCTCTGCTGGTGGCCCTGCTGCTCCTGAGCCTGGCCTTGGAGACCCAGCAGGCGGACAGCGGCGGCTACGACCCCGCCCCGACGCCGCTCACCACCTCCCACAGCAACAGGAAGCCTCCCAGGCCGCGTCCGCATCCGCCGCGCCCTCGCCCTCGCCCTCGCCCCCGCCCACCTACACCTCCCACGCCAACGCCGCCGCCGTACACTCCGTCCCCTCCTGAGCCTGAACCTACGCCGCCCCCGTGCACTCCGTCCCCTCCTGAGCCTGAACCCACTCCGTCCCCTCCTGAGCCTGAGCCTGAGCCCACGCCAACG

>**e,** BJ250155 Triticum aestivum cDNA clone whf9f23 **3'**, mRNA sequence

CAACAGTACGTAGCAATATCACGGGTAACCAGCAATCTGCATTATTATTCAGACTACATATTACTTAGATCTCCGATGAGAATGGCCCTGCATGCGTTGGCAGGGACTCATAGCCACAGCCCTAGACATTGGGCTTGTAGGCAGGCGTCGATGGCGGCTCCTCGTAGTCGGCCGCGTCGGCGGCAGCCTGTACGCCAGCGGAGTCGGCTTCCGCGCCGTGGTCGAGGAAGGGAGATGGCATCGGGGCCGGGGCGTATGGGGGTGGCGTGGGCTCAGGAGGGGACGGAGGGGACGGAGTGCACGGCGGCGGCGTTGGCGTGGGCTCAGGCTCAGGCTCAGGAGGGGACGGAGTGGGTTCAGGCTCAGGAGGGGACGGAGTGTACGGGGGCGGCGTAGGTTCC

>**f,** Complimentarily reversed BJ250155

GGAACCTACGCCGCCCCCGTACACTCCGTCCCCTCCTGAGCCTGAACCCACTCCGTCCCCTCCTGAGCCTGAGCCTGAGCCCACGCCAACGCCGCCGCCGTGCACTCCGTCCCCTCCGTCCCCTCCTGAGCCCACGCCACCCCCATACGCCCCGGCCCCGATGCCATCTCCCTTCCTCGACCACGGCGCGGAAGCCGACTCCGCTGGCGTACAGGCTGCCGCCGACGCGGCCGACTACGAGGAGCCGCCATCGACGCCTGCCTACAAGCCCAATGTCTAGGGCTGTGGCTATGAGTCCCTGCCAACGCATGCAGGGCCATTCTCATCGGAGATCTAAGTAATATGTAGTCTGAATAATAATGCAGATTGCTGGTTACCCGTGATATTGCTACGTACTGTTG

**Supplementary Fig. 1. Sequences from public data used to verify and reconstruct the *TaPGW1-A1*** **gene. a,** NT sequence of *TraesCS6A02G174100LC*. **b,** Protein sequence of TraesCS6A02G174100LC. **c,** Comparison of protein sequences TraesCS6A02G174100LC and *At*ERF. **d,** NT sequence of cDNA *BM135088*. **e,** NT sequence of cDNA *BJ250155*. **f,** Reversed NT sequence of cDNA *BJ250155*. The sequences highlighted in green overlap.

>**a,** TaPGW1-A1 exon length=927 nt

TCCCAGAACCACACCGATCGAGCCAAGAACTTTGCAGTGCGTGAGGCGGTGAGGGAGTGCGAGAATGGCGGGTGGCAAAGCCGCTCTGCTGGTGGCCCTGCTGCTCCTGAGCCTGGCCTTGGAGACCCAGCAGGCGGACAGCGGCGGCTACGACCCCGCCCCGACGCCGCTCACCACCTCCCACAGCAACAGGAAGCCTCCCAGGCCGCGTCCGCATCCGCCGCGCCCTCGCCCTCGCCCTCGCCCCCGCCCACCTACACCTCCCACGCCAACGCCGCCGCCGTACACTCCGTCCCCTCCTGAGCCTGAACCTACGCCGCCCCCGTACACTCCGTCCCCTCCTGAGCCTGAACCCACTCCGTCCCCTCCTGAGCCTGAGCCTGAGCCCACGCCAACGCCGCCGCCGTGCACTCCGTCCCCTCCGTCCCCTCCTGAGCCCACGCCACCCCCATACGCCCCGGCCCCGATGCCATCTCCCTTCCTCGACCACGGCGCGGAAGCCGACTCCGCTGGCGTACAGGCTGCCGCCGACGCGGCCGACTACGAGGAGCCGCCATCGACGCCTGCCTACAAGCCCAATGTCTAGGGCTGTGGCTATGAGTCCCTGCCAACGCATGCAGGGCCATTCTCATCGGAGATCTAAGTAATATGTAGTCTGAATAATAATGCAGATTGCTTGGTTACCCGTGGATATTGCTACGTACTGTTGTGTTACTGTAAAACCATTATCTATATATAATATGGAAATGGAAATGGGAAACTGCATGTGTTGTGTCCTCTTCCTCCGTTGTTCAACTTCAACAGCATGAGTTCTTTGCATGTAAAAACAGTAAATACTATGGCAAAACCATGTTCTGCAGGATGCCCATGCCACGCTTGAACAAATACGTAAGATCAAGTCAGCACCGTGGCGCACGCTAAGTTGCC

>**b,** *Ta*PGW1-A1 protein sequence length=173 aa

MAGGKAALLVALLLLSLALETQQADSGGYDPAPTPLTTSHSNRKPPRPRPHPPRPRPRPRPRPPTPPTPTPPPYTPSPPEPEPTPPPYTPSPPEPEPTPSPPEPEPEPTPTPPPCTPSPPSPPEPTPPPYAPAPMPSPFLDHGAEADSAGVQAAADAADYEEPPSTPAYKPNV*

```
>c, XBH77282 Hypothetical protein VPH35_103785 [Triticum aestivum] (T2T)
MAGGKAALLVALLLLSLALETQQADSGGYDPAPTPLTTSHSNRKPPRPRPRPRPRPRPPTPPTPTPPPYTPSPPEPEPTPPPYTPSPPEPEPTPSPPEPEPEPTPTPPPCTPSPPSPPEPTPPPYAPVPMPSPFLEHGAEADSAGVQAAADAADYEEPPSAPAYKPIV
>d, Comparison of protein sequences
TaPGW1-A1                    MAGGKAALLVALLLLSLALETQQADSGGYDPAPTPLTTSHSNRKPPRPRPHPPRPRPRPR  60
                             MAGGKAALLVALL LSLALETQQADSGGYDPAPTPLTTSH NRKPPRPRPHPPRPRPRPR
Extensin (XP_037455269)      MAGGKAALLVALLFLSLALETQQADSGGYDPAPTPLTTSHRNRKPPRPRPHPPRPRPRPR  60

TaPGW1-A1                    PRPPTPPTPTPPPYTPSPPEPEPTPPPYTPSPPEPEPTPSPPEPEPEPTPTPPPCTPSPP  120
                             PRPPTPPTP PPPYTP              SPPEPEPTP P  PEPEPTPTPPP TPSPP
Extensin (XP_037455269)      PRPPTPPTPMPPPYTP--------------SPPEPEPTPPPSPPEPEPTPTPPPYTPSPP  106

TaPGW1-A1                    SPPEPTPPPYAPAPMPSPFLDHGAEADSAGVQAAADAADYEEPPSTPAYKPNV  173
                             SPPEPTPPPYAP PMPSPFLDHGAEADSAGVQAAADAADYEEPPSTPAYKPNV
Extensin (XP_037455269)      SPPEPTPPPYAPVPMPSPFLDHGAEADSAGVQAAADAADYEEPPSTPAYKPNV  159
```

**Supplementary Fig. 2. Sequence comparison of *Ta*PGW1-A1, XBH77282 and XP_037455269. a,** NT sequence of *TaPGW1-A1*. **b,** Protein sequence of *Ta*PGW1-A1. **c,** Protein sequence of XBH77282. **d,** Comparison of the protein sequences of *Ta*PGW1-A1 and XP_037455269.

**Supplementary Fig. 3. Characterization of *Ta*****PGW1-A1 protein. a,** Signal peptide prediction. The predicted signal peptide sequence region (1-26 a.a.) and its cleavage site are indicated. **b,** Hydrophobicity analysis. The x‑axis represents the amino acid sequence position; the y‑axis represents the hydrophobicity score. In the figure, peaks above the threshold (0) represent hydrophobic regions, whereas regions below the threshold represent hydrophilic regions. **c,** NLS prediction. The predicted NLS core sequence and its position are marked.

**Supplementary Fig. 4. Phenotypic analyses of *TaPGW1-A1* overexpression plants. a,** PCR amplification of the *Ubi+GUS* fragment using vector-specific primers. **b,** PCR amplification using the *TaPGW1-A1* upstream primer and a vector-specific downstream primer for verification of exogenous transgene integration. The wild type (WT) was used as the negative control (-), and the plasmid vector was used as the positive control (+). **c,** Copy number of *TaPGW1-A1* in transgenic positive lines. **d,** Transcript levels of *TaPGW1-A1* in transgenic positive lines. OE1, OE2, and OE3 represent three independent transformation events; WT denotes the wild type. **e-h,** Comparison of 1,000-grain weight in 2022-2023 (e) and in 2023-2024 (f), grain length (g), and grain width (h) between WT and OE1-OE3 plants. **i-k,** Comparison of spikelet number (i), spike length (j), and grain number per spike (k) between WT and OE1 plants under field conditions. Statistical analyses were performed using R (version 4.3.2) for Duncan’s multiple range test, and graphs were generated with GraphPad Prism 8. Different letters above bars indicate significant differences at α=0.05 according to Duncan’s multiple range test.

**Supplementary Fig. 5. Endosperm cellularization in *TaPGW1-A1* overexpression transgenic wheat. a,** Cross-sections of developing grains from WT and OE1-OE3 lines at 3, 6 DPA. Scale bar, 200 μm. **b,** Quantitative analysis of the number of endosperm cell nuclei in grain cross-sections of WT and OE1-OE3 lines at 6 DPA. Statistical analyses were performed using R (version 4.3.2) for Duncan’s multiple range test, and graphs were generated with GraphPad Prism 8. Different letters above bars indicate significant differences at α=0.05 according to Duncan’s multiple range test.**Supplementary Fig. 6.** **RNA-seq analyses of *TaPGW1-A1* overexpression lines. a,** Principal component analysis (PCA) of the RNA-seq samples. **b,** qRT-PCR validation of selected differentially expressed genes. Statistical analyses were performed using R (version 4.3.2) for Duncan’s multiple range test, and graphs were generated with GraphPad Prism 8. Different letters above bars indicate significant differences at α=0.05 according to Duncan’s multiple range test.

**Supplementary Fig. 7. *Ta*PGW1-A1 fragments and subcellular localization. a,** Five *Ta*PGW1-A1 truncations used in the Y2H assays. **b,** Two truncations used for subcellular localization analysis. **c-f,** Transient expression of GFP control (c), *Ta*PGW1-A1-C-GFP (d), *Ta*PGW1-A1-GFP (e), and *Ta*PGW1-A1-N2-GFP (f) in *N. benthamiana* epidermal cells. Scale bar, 20 μm.

**Supplementary Fig. 8. BSMV-mediated editing of *TaDSK2a.* a,** Two sgRNA sequences for gene editing. **b, c,** Leaf phenotypes: normal before BSMV infection and viral symptoms after BSMV infection. **d,** Sequencing Chromatograms of unedited *TaDSK2a*. The sgRNA-2 binding site is highlighted in yellow. **e-g,** The impact of *TaDSK2a-1A*, *TaDSK2a-1B*, and *TaDSK2a-1D* editing types on the primary structure of the encoded proteins. Black arrows denote premature translation termination.

**Supplementary Fig. 9. Phenotypic analysis of edited *TaDSK2a* plants**. **a,** 1,000-grain weight, **b,** Grain width. **c,** Grain length. Statistical analyses were performed using R (version 4.3.2) for Duncan’s multiple range test, and graphs were generated with GraphPad Prism 8. Different letters above bars indicate significant differences at α=0.05 according to Duncan’s multiple range test. d1–d5 represent five edited genotypes: d1 and d2, homozygous edits in *TaDSK2a-1A*; d3, a homozygous edit in *TaDSK2a-1B*; d4, a homozygous edit in *TaDSK2a-1D*; and d5, homozygous edits in *TaDSK2a-1A* and *TaDSK2a-1B*.

**Supplementary Fig. 10. Y3H assays for *Ta*PGW1-A1, *Ta*RGLG2, and *Ta*DSK2a.** The following construct combinations were tested: AD and *Ta*RGLG2-BD; *Ta*PGW1-A1-AD and RGLG2-BD; *Ta*PGW1-A1-AD and *Ta*RGLG2-*Ta*DSK2a. Transformants were spotted on SD/-Leu/-Met/-Trp and SD/-His/-Leu/-Met/-Trp media. Transformants were serially diluted (10^0^-10^-3^).

>*TaPGW1-B1* chr6B: 159179275-159180122 (- strand) length=848

CAGAACTAGCTTTGGTTGCCATAGTATTGACTGTTTCGACATGCAAACATCACATCTCATCAGCGGAACTCATGTTATTGACGTTGAACTCATGTTGGGACAAACAACATGCAGTTTCCCATCTCCATTTCCACATTATACTATCAGATAATGGTTTTACAGTAACACCACAACAGTAGCAATACCCACATGCATGAAAGTACGCTAACTCTGGACGAACACAAATCAGCGGCGGGTAACCAAGCAATCTGCATTATTATTCAGACTACATATTACTTAATCTCCGAGAGAATGGCCCTGCATGCATTGGCAGGGACTCACGGTCACAGCCCTAAGCATTATCCGGCGTCGATGGCGGCTCCTCGTAGTCGGCGGCAGCCTGTACGCCAGCGGAGTCGGCTTCCACGCCGTGCTCGAGGAAGGGAGATGGCATCGGGGCCGGGGCGTATGGGGGTGGCGTGGGCTCAGGAGGGGACGGAGTGTACGGCGGCAGTATTGGCGTGGGCTCAGGAGGGGACGGAGTGTACGGCGGCGGCGTTGGCGTGGGCTCAGGAGGGGACGGGGTGTACGGGGGCAGCGTTGGCGTGGGAGGGCGCGGCGGAGACGGACGCCGCTTCCTGTGGGACGTGGTGAGCGGCGTCGCCGTCGGGGCGGGGTCGTAGCCGCCGCTGTCCGCCTGGGTCTCCAAGGCCAGGCTCAGGAGCAGCAGGGCCACCAGGAGAGCTGCTTTGCCACCCGCCATTCTCTCACTCCCTCACGCACTGCAAAGTTCTTGGCTCGATCGGTGTGGTTCTGAGAAGATGGGTGTAACCATGCCGCTGCTTATATACACGGAGGGAGGAGTCGAG

>*TaPGW1-D1* chr6D: 83255201-83256425 (- strand) length=879

CCAATCCAACCTCCCCCGCATGCCTAGCGCCAGCACCATCGCTTGCGCGCACAAGCAAGGACCCCTCACTCCGTGAATCCGTGTATATAAGGAGTCGTATGGGCTCACCCATCTTCTCATAACCACACCGATCGAGCCAAGAACATTGCAGTGCGTGAGGGAGTGAGAGAATGGCGGGTGGCAAAGCCGCTCTCCTGGTGGCCCTGCTCTTCCTGAGCCTGGCCTTGGAGACCCAGCAGGCGGACAGCGGCGGGTACGACCCCGCCCCGACGCCGCTCACCACGTCCCACAGGAAGCGGCGTCCGTCTCCGCCGCGCCCTCGCCCCCGCCCACCTACACCTCCCACGCCAACGCCGCCCCCGTACACTCCGTCCCCTCCTGAGCCTGAACCCACGCCGCCCCCGTCCCCTCCGTCCCCTCCTGAGCCTGAGCCCACGCCAACGCCGCCGCCGTACACTCCGTCCCCTCCTGAGCCCACGACACCCCCATACGCCCCGGCCCCGATGCCATCTCCCTTCCTCGAGCACGGCGCGGAAGCCGACTCTGCTGGCGTACAGGCCGCCGCCGACGCCGACCTACAAGCCCAGAGTCTAGGGCTGTGAGTCCCTGGCAACGCATGCAGGGCCATTCTCATCGGAGATCTAATAGTATGTAGTCTGAATAATAATGCAGATTGCTTGGTTACCCGCTGATGATTTGTGTGCGTCCAGAGTTAGCGTACTATTATGTGGATGTGGGTGTTGCTACTGTTGTGGTGTTACTGTAAAACCATTGTCTGCTATAATATGGAAATGGAAATGGGAAACTGCATGTGGTGTGTCCTCTTCCTCCGTTGTTCAACTTCAACAGCATGAGTTCTTTGCATGTCGAAACAGTAAA

>*TaPGW2-A1* chr6A: 100675348-100676443 (+ strand) length=1096

AAGCAAAGGACTCGACTCCTCCCTCCGTGTATATAAGCAGCGGCGTGGTTACACCCATCTTCCCAGAACCACACCGATCGAGCCAAGAAGTTTGCAGTGCGTGAGGCGGTGAGGGAGTGCGAGAATGGCGGGTGGCAAAGCCGCTCTGCTGGTGGCCCTGCTGCTCCTGAGCCTGGCCTTGGAGACCCAGCAGGCGGACAGCGGCGGCTACGACCCCGCCCCGACGCCGCTCACCACCTCCCACAGCAACAGGAAGCCTCCCAGGCCGCGCCCTCGCCCTCGCCCTCGCCCCCGCCCACCTACACCTCCCACGCCAACGCCGCCGCCGTACACTCCGTCCCCTCCTGAGCCTGAACCTACGCCGCCCCCGTACACTCCGTCCCCTCCTGAGCCTGAACCCACTCCGTCCCCTCCTGAGCCTGAGCCTGAGCCCACGCCAACGCCGCCGCCGTGCACTCCGTCCCCTCCGTCCCCTCCTGAGCCCACGCCACCCCCGTACGCCCCGGTCCCGATGCCATCTCCCTTCCTCGAGCACGGCGCGGAAGCCGACTCCGCTGGCGTACAGGCCGCCGCCGACGCCGCCGACTACGAGGAGCCGCCATCGGCGCCGGCCTACAAGCCCATTGTCTAGGGCTGTGACTGTGAGTCCCTGCGAACGCATGCAGGGCCATTATTCTCATCGGAGATCAAATACTAATATGTAGTCTGAATAATAATGCGGATTGCTTGGTTACCCGCTGCTGATTTGTGTTCGTCCAGAGTTAGCGTACTTTCATGCATGTGGGTATTGCTACTGTTGTGGTGTTACTGTAAAACCATGAAATGGAAATGGGAAACTGCATGTGGTTTGTCCTCCGCTGTACGTATGTTCAACTTCAACAACATGAGCTCTTTCGCTGAGGAGATGTGATGTTTCCATGTGAAACAGTAAATAAGACTATGGCAACCAAAGCTAGTTCTGCAGGATGCCCATGCCACGCTTGAACTGGCTTGAACAAATACGTGCGATCAAGCTCTTTAGCTGAGCGCACCGTGGCGCATGCCAACCGTCCGATCAACGTTTATCCTTTTTTTTTATCTCACATCCCGATGAACG

>*TaPGW2-B1* chr6B: 159311079-159311612 (+ strand) length=534

ATGGCGGGTGGCAAAGCCGCTCTCCTGGTGGCCCTGCTGTTCCTGAGCCTGGCCTTGGAGACCCAGCAGGCGGACAGCGGCGGGTACGACCCCGCCCCGACGCCGCTCACCACGTCCCACAGGAACAGGAAGCCTCCCAGGGCGCGTCCGCATCCGCCGCGCCCTCGCCCTCGCCCTCGCCCCCGCCCACCTACACCTCCCACGCCAATGCCGCCCCCGTACACTCCGTCCCCTCCTGAGCCTGAACCCACGCCGCCACCGTCCCCTCCTGAGCCTGAGCCCACGCCAACGCCGCCGCCGTACACTCCGTCCCCTCGTCCCCTCCTGAGCCCACGCCACCCCCATACGCCCCTGTCCCGATGCCATCTCCCTTCCTCGACCACGGCGCGGAAGCCGACTCCGCTGGCGTACAGGCCGCCGCCGATGCCGCCGACTACGAGGAGCCGCCATCGACGCCGGCCTACAAGCCCAATGTCTAGGGCTGTGACTGTGGGTCCCTGCCAACGCATGCAGGGCCATTCTCTCGGAGATTAA

>*TaPGW2-D1* chr6D: 83444920-83445691 (+ strand) length=772

CCCAGAACCACACCGATCGAGCCAAGAAGTTTGCAGTGCGTGAGGGAGTGAGAGAATGGCGGGTGGCAAAGCCGCTCTCCTGGTGGCCCTGCTCTTCCTGAGCCTGGCCTTGGAGACCCAGCAGGCGGACAGCGGCGGGTACGACCCCGCCCCGACGCCGCTCACCACGTCCCACAGGAAGCGGCGTCCGTCTCCGCCGCGCCCTCGCCCTCGCCCTCTCCCCCGCCCACCTACACCTCCCACGCCAACGCCGCCCCCGTACACTCCGTCCCCTCCTGAGCCCACTCCGCCCCCATACATTCCGTCCCCTCCTGAACCCACGCCACCCCCATACGCCCCGGCCCCGATGCCATCTCCCTTCCTCGAGCACGGCGCGGAAGCCGACTCCGCTGGCGTACAGGCCGCCGCCGACGCCGCCGACTATGAGGAGCCGCCATCGACGCCGCCGGCCTACAAGCCCAATGTCTAGGGCTGTGACTGCGAGTCCCTGCCAACGCATGCAGGGCCATTATTCTCATTGGAGATTAAGTAATATGTAGTCTGAATAATAATGCAGGTTGCTTGGTTACCCGCTGCTGATTTGTGTTCGTCCAGAGTTAGCGTGCTATCATGCCCGTGGGTCTTTGTCGAAAAAATCATGCATGTGGGTTTTGCTACCGTTGTGGTGTTACTGTAAAACGTAGCATTGTCTACTGAATCTGTCGTGTGTCCTCCGCTGTTTAACTTCAACACGAGCATGAGTTCTTTCGCTGATGAGATGTGATATGTTTGC

**Supplementary Fig. 11. Sequences and physical locations of *TaPGWs*.** The physical locations were determined based on IWGSC RefSeq v1.1 genome. A gap in *TaPGW2-D1* was filled using RNA-seq data.

**Supplementary Fig. 12. Proposed working model for the *Ta*PGW1-A1**–***Ta*DSK2a**–***Ta*RGLG2** **regulatory module.** *Ta*PGW1-A1 co-localizes with *Ta*DSK2a at the cell periphery during early grain development. *Ta*DSK2a recognizes K48- and K63-linked polyubiquitin chains and may regulate selective protein turnover and ubiquitin-mediated signaling. Interaction analyses suggest that *Ta*RGLG2 may modulate the *Ta*PGW1-A1–*Ta*DSK2a interaction. These activities are proposed to influence the abundance or activity of cell cycle-related factors, including Cyclin-L, THP, and KIN-12F, thereby delaying endosperm cellularization, prolonging the proliferative phase of free nuclei, increasing cell number, and ultimately enhancing grain size and grain weight. Solid arrows indicate direct actions, dashed arrows indicate potential pathways, and T-shaped dotted lines indicate inhibitory effects.

**Supplementary** **Fig. 13. Schematic representation of the overexpression vector for *TaPGW1-A1*.** The *ActinI*::*TaPGW1-A1*::*Ter* expression cassette was inserted into the pUCBS vector skeleton to generate an expression construct.
